# Impacts of Professionalization and Wellbeing Policies on Scottish Prison Workers

**DOI:** 10.3389/fsoc.2021.757583

**Published:** 2021-10-25

**Authors:** Andrew Fletcher, Linda McKie, Isobel MacPherson, Jackie Tombs

**Affiliations:** ^1^ Department of Philosophy, Durham University, Durham, United Kingdom; ^2^ School of Social and Political Science, The University of Edinburgh, Edinburgh, United Kingdom; ^3^ Community Health Research and Evaluation, Glasgow, United Kingdom; ^4^ Department of Social Sciences, Glasgow Caledonian University, Glasgow, United Kingdom

**Keywords:** discretion, “policy and practice”, prison work, professionalization, wellbeing, “whole-prison approach”

## Abstract

Prison workers occupy a niche role. Balancing the care and welfare of prisoners while simultaneously restricting their freedoms is a stressful job, laced with danger, that occurs entirely within the bounded context of the prison. Here, wellbeing and professionalism are closely linked and articulated through a range of policies. This article explores the perceptions and experiences of staff in relation to a range of wellbeing and training policies in the Scottish Prison Service (SPS). We interviewed 10 SPS employees, some working directly with prisoners and others in more centralised policy development and support roles. Thematic analysis found a high degree of contentment with such policies but highlighted tensions between their implementation and specific challenges of the prison context. Emerging themes included: supporting wellbeing within the complex dynamic of the prison world; addressing inherent tensions borne out of the underlying threat of violence; and the impact of professionalization. We conclude that while the prison service aspires to offer employees wellbeing and professionalization opportunities similar to those in other sectors, there is a need for such policies to more clearly reflect the unique context of prison work. This might involve co-design of policies and more careful consideration of the pressures, tensions and idiosyncrasies of that rarefied environment.

## Introduction

Prison workers’ experiences receive little attention in mainstream work and employment literature, although the roles of probation officers and ex-offenders, and their interactions with wider society have had some critical examination (Gale 2012; Kirton and Guillaume 2019). In-prison work is even considered “dirty work” by some (Hughes et al., 2017: 108) and is comparatively marginalised among studies of different occupations. However, prison staff face a unique set of ideological tensions and pragmatic hazards in their daily roles, and “should be seen as a distinct occupational group … worthy of study in their own right” (Bennett et al., 2013: 2). Media reports often focus on declining safety and increased anxiety and depression among prison staff, framed as an outcome of austerity measures (among many examples, see BBC 2020). While limited resources remain a significant issue for prison services, the social dynamics and relationships between prisoners and prison workers, and the policy frameworks that govern their behaviours, are complex.

Criminal justice and profession-specific journals have given some attention to prison workers’ wellbeing, including their experiences of work-related stress (Carlson, Anson, and Thomas 2003; Harvey 2014; Lovell and Brown 2017; Ricciardelli, Power, and Medeiros 2018; Steiner and Wooldredge 2015), work-life balance (Kinman et al., 2017) and impacts of the job role on family relationships (Akoensi 2018).^1^ The most prominent study of UK prison workers’ wellbeing is Liebling’s longitudinal quality of life survey, which has been generating data since 2006 (Liebling and Arnold 2004; Liebling, Price, and Shefer 2011: 210). As part of this, a baseline study in HMP and YOI Grampian, the UK’s first “community-facing” prison, found low staff wellbeing, poor organisational structure and leadership, and unclear boundaries between staff and prisoners, in spite of its innovative policies and practices (Schmidt et al., 2015). However, the one-year follow-up report found a more stable regime, with more confident and competent staff: “*Make use of staff qualifications! We’re multi-skilled and have lots to offer*” (Schmidt et al., 2017: 1). While a positive development, prison workers cultivate a unique set of skills through their work and need to be empowered to put these to best use; their role is far more than that of a turnkey.

Recent Scottish Government and SPS policy initiatives aim to take a more “holistic” approach to prison staff safety and wellbeing, and to “professionalize” their job roles. This “whole prison approach” (SPS 2014) has been generally well received but has also revealed some of the specific tensions that emerge in the unique context of prisons. For example, when prison workers must decide when and how to enforce discretionary policies; to find a balance between engaging with prisoners and keeping a professional distance; or to sacrifice some of their own freedoms due to working in a secure environment. Similar tensions are inherent in the work of “street-level bureaucrats,” defined as a subset of civil servants who have direct contact with the general public (Lipsky 1969). While prison work might not involve working with “the general public,” there are significant overlaps in the challenges faced by prison workers and street-level bureaucrats, including: use of psychological capital in the face of increasing “red tape” (Dudau, Kominis, and Brunetto 2020); professional values under new managerialism (Jacobsson, Wallinder, and Seing 2020); and identities and “professionalization” (McCann and Granter 2019).

This article uses data from an ESRC-funded study that explored the intersection of policy and workplace realities for a range of prison service employees in terms of their wellbeing and professionalization.^2^ The *analysis* was conducted under the ERC-funded “Knowledge for Use” project (see acknowledgment).

### Linking Wellbeing and Professionalization

Prisoner officers and prisoners are in close physical and psychological proximity. Their relationships are built around a complex set of interdependencies, and their mental and physical wellbeing are closely linked (Beynon and Drew 2005). Officers have the power to grant or withhold perks or freedoms but simultaneously require prisoners to cooperate with specific safeguarding and disciplinary regulations, creating a positive feedback loop: contented prisoners lead to a safer working environment and increased overall wellbeing. Policymakers understand this; in-prison wellbeing promotion is often aimed at both staff and inmates. For example, smoking cessation programmes, which affect both groups in complex ways (Brown et al., 2019). Like islands, prisons are somewhat isolated from wider society. They evolve their own ecosystems; social processes, economies and groupings that might echo those seen in the ‘outside world’ but are governed by the organisation and regime of the prison. Most prisoners and all prison staff must transition between the “inside” and “outside” worlds, with inevitable consequences on their expectations and perceptions of workplace reality and stress levels (Thompson 2000).

This high-pressure environment, combined with the underlying threat of physical and/or psychological violent conflict, is one of the most significant factors affecting prison staff wellbeing (Liebling, Price, and Shefer 2011: ch.4). A review of research into the factors underlying violence in prisons concluded that the physical environment, and its routines and cultures, plays a key role. Other significant factors included notions of justice and fairness, purposeful activities and, crucially for this article, the availability and skills of unit staff (McGuire 2018). Well-trained and experienced staff minimise violence in prisons and the accompanying stress this brings. This intrinsic link between staff and prisoner wellbeing is reflected in the linked wellbeing and professionalization agendas—the “whole prison approach”—within SPS policy.

Castle (2008) found prison officers’ job satisfaction to be positively correlated with supervisory support and negatively correlated with education level. This was attributed to a “lack of opportunities for advancement and promotion in correctional work … The jail officer position is typically one of low salary and prestige” (ibid.: 58). At that time, the overall picture was of a low-status job that required specialist skills, which were only gradually becoming formalised. More recently, Kinman et al., 2019 identified high levels of presenteeism among prison officers, underpinned by policy-related (punitive absence management systems), solidarity-related (fear of letting colleagues down) and career-related (sense of duty and professionalism) themes. Liebling, Price, and Shefer (2011) connect the shift towards professionalization in the prison service to the May Report in 1979, which identified a need for modern management approaches. These brought about the development and use of performance management and Key Performance Indicators (KPIs), which some prison staff believe *…fail to capture the essence of their work* (quoted in Liebling, Price, and Shefer 2011: 202), so the tendency is *to do what is inspected not what is expected* (ibid.: 200).

The push towards professionalization has also highlighted the role of training in instilling organisational values, decision-making skills, competencies and codes of practice. In her study of Scottish Prison Officers’ induction training, Morrison (2019: 25) observes that “…the policy-heavy elements of the training had little impact on [recruits] and were unlikely to have any enduring effect.” The trainees saw the e-learning system (used for the policy elements) as a tick-box exercise that was more about protecting the organisation than embedding learning. Unsurprisingly, much of the training (at induction and ongoing) is directed at reducing inherent risks and is delivered alongside more generic wellbeing initiatives for both staff and inmates. Tensions between the organisational desire for professionalization and the training underpinning it, and the realities of daily work in the prison service and its impact on wellbeing, are not addressed in the literature.

### The Prison Service Context in Scotland

The Scottish Prison Service (SPS) is an Executive Agency of the Scottish Government. It has overall responsibility to promote health and wellbeing, and to ensure that all employees have fair and equitable access to wellbeing activities and resources. This agenda is driven by a “whole prison approach,” central to which is the importance of prisoner involvement and feedback, and “the role prison staff can play in having a positive impact on health and wellbeing” (SPS 2014: 37). The SPS *Employee Wellbeing* document emphasises “fostering a positive working environment … through appropriate wellbeing activities and resources” and incorporates the Scottish Centre for Healthy Working Lives Award Programme, which encourages employees “to take personal responsibility for their own health, safety and wellbeing, whilst also ensuring that the safety of their colleagues and others in the workplace is safeguarded” (SPS 2018: 1). Building on this, the Health and Social Care in Prisons Programme Board was established to develop integrated health and social care models for the prison service (SPS 2019b: 12). For example, given the rising absentee rates linked to stress, “Absence Management” strategies relating to “mentally healthy workplaces” have introduced “targeted intervention strategies to address specific localised challenges” (ibid.: 9).

In the same period, *Forward Together: SPS Vision, Mission and Values Partnership Agreement* (2016a), was designed to create “a degree of skill and professionalism that [had] not before been made available in the prison setting” (SPS 2016b: 1). The SPS “…will establish an expanded role with greater impact for prison officers [who] will get the opportunity and the skills to influence and change lives: as counsellors, role models, coaches and advocates of the people in their care” (ibid.: 23). The changes would “empower, motivate and equip” frontline staff and enhance “job satisfaction through professional recognition and associated reward” (ibid.: 28).^3^

The SPS clearly recognises the wide range of social responsibilities encompassed in prison work and that these responsibilities must be properly supported with appropriate training. But external pressures on the service, such as increasing prisoner numbers, make it difficult for workers to absorb this extra training despite its good intentions. The reality on the frontline is that there were 4,477 staff employed in the SPS as of March 2019, and 8,267 inmates (a rise of 700 inmates over the previous year) as of August 2019. This was due in part to an increased focus on prosecuting serious organised crime, sexual violence and domestic abuse cases. The increased pressure has resulted in:

• The SPS exceeding its operating capacity of 7,676 (and approaching its maximum capacity)
• High sickness absence rates with almost twice the average days lost (17 days) compared with England and Wales (9.3 days)
• A 32% increase in stress-related absence from 2017 to 18 to 2018–19
• Prison officers working increased hours to cover absences
• A decline in meaningful activity for inmates due to staff absences and lack of space
• A lack of single cells to let inmates cool off or to separate serious crime rivals
• An increase in all categories of assault except serious assaults on staff (Auditor General for Scotland 2019).

A recent critical report attributed the rise in drug-related crimes, poor conditions and increased segregation of prisoners to understaffing (Council of Europe 2019). While beleaguered by increasing pressures and shifting dynamics, the SPS is making efforts to address overall health and welfare, and managers recognise the interdependence of prisoner and staff wellbeing. The policy context is becoming more supportive, with a dual focus on wellbeing and professionalization, but there remains a need to design and implement comprehensive policy frameworks that can accommodate the realities of prison work. This article therefore sets out to explore wellbeing and professionalization policies closer to the front line.

## The Views of Prison Workers

We interviewed 10 SPS staff, using a semi-structured schedule, to explore their perceptions of developing and using policies in everyday prison work. Five participants worked at the prison service headquarters, where they had a deeper insight into the origins of organisational policy, and five worked in prisons, where they saw the implementation and effects of policies. We wanted to capture the voices of allied specialist staff (not “rank and file” officers, or managers and policymakers), which often go unheard in social research. All participants had experience working with prisoners as well as additional policy-related training associated with their specialist role. The HQ staff roles included health provision, procurement, security, finance, administration and legal services, while the in-prison support staff roles covered intervention and rehabilitation, staff training, finance and compliance, inmate finance, and security. This range of perspectives highlighted a diversity of experiences across the service. Due to our use of convenience sampling and the level of reassurance we were required to give to protect both the SPS and individuals, only 10 staff were forthcoming. While this small sample size precludes us from asserting our findings more generally, our conclusions indicate a set of policy-related issues worth exploring in more depth.

The interviewees had a range of experience; the newest employee had worked for the SPS for 2 years, while the most experienced had worked there for 23 years. We recognise that frontline experience and formal training are not the same, and equip workers with different skills bases and attitudes. Retrospectively, we noticed that those who had worked in prisons for longer seemed more comfortable talking about their use of “discretion,” whereas newer staff tended to rationalise their front line experiences within the terms of the training they had received. Experience vs training is a complex issue worth exploring, but our data indicated a more policy-focussed angle.

Ethical approval was obtained from the relevant committee of the lead university, and access negotiated with SPS senior management and local prisons. Pseudonyms are used to protect anonymity, while specific job roles have been generalised to either “prison-based” or “headquarters-based.” Interview transcripts were analysed using NVivo (qualitative data analysis software) and a thematic coding schedule was developed, revealing common and specific experiences among all interviewees. Validation was undertaken by sharing the analysis and themes with the interviewees, who had the opportunity to give feedback or clarification.

Three broad themes were identified:

• Supporting the equal provision of wellbeing initiatives in the context of differing work demands and challenges, which focused on flexibility and training.
• Addressing the inherent tensions within prisons, such as the underlying threat of violence and achieving the balance between care and punishment.
• The impact of professionalization on wellbeing and the everyday experiences of prison workers, including longer-term career implications.

### Supporting Wellbeing Within the Prison Service

The SPS’s wellbeing agenda is underpinned by its “whole prison approach.” Respondents appreciated this proactive and integrated ethos, with its focus on involvement.

Management and the hierarchy seem to be more interested in staff welfare and stuff like that. I cannae fault them for that. A lot of initiatives are about trying to get staff [involved]. If they don’t know about it themselves, they’ll try and give them the information and try and make it better. (Peter, Prison staff, 5 years in service)

We try to put a lot of things in place just now about Healthy Working Lives and there’s a committee set up and they’re trying to come up with alternatives… We have people coming to do health monitor check-ups. It’s totally voluntary but it’s quite good. (Gary, HQ staff, 18 years)

While some staff felt they could see its underlying drivers, most felt positive about the wellbeing agenda.

It costs you less to keep a member of staff at their work than being off sick, doesn’t it? I’m maybe cynical about things but I believe that, yeah, there’s a genuine desire to do that but also when you look at the cost efficiency and stuff like that, it’s a lot easier to do that, to prevent things than fix them. (Peter, Prison, 5 years)

However, it became clear that creating and enacting a wellbeing agenda that worked equally well across the service was problematic. The ability to access wellbeing initiatives depends on which part of the prison service you work in. For example, many of the prison-based staff work shifts and, for those working antisocial hours, some initiatives are not available. The impact of shift work also extends to diet and eating habits.

In prison, it’s shifts… and limited availability of fresh food. [It’s] vending machines out of hours. (Linda, HQ, 9 years)

In the health sector, the NHS Five Year Forward View pledged to make nutritious (not junk) food available for all hospital staff, especially shift workers, and moreover noted that employers are the key to promoting healthier lifestyles (NHS England 2014: 11–12).^4^ Similarly, wellbeing activities that can be organised at headquarters (Pilates classes, walking groups) are more difficult in the prison building itself, which may have limited space or facilities, or restrictions on what types of service can or cannot be brought onsite.

When you’re working there, basically you are a prisoner as well. It’s not like you can nip across to Marks and Sparks and get a cup of coffee. (Glen, HQ, 23 years)

Aside from requiring time and flexibility, staff must *want* to take up these opportunities. Initiatives can start off on a wave of enthusiasm, which gradually fades to the committed and those whose work-life balance allows them to participate. Self-motivation shifts responsibility onto the individual but when their ability to participate is determined by external factors, such as caring roles or inflexible working patterns, it becomes an issue of accessibility, highlighting a weakness in the initiative. Respondents emphasized the importance of flexibility in maintaining both individual wellbeing and that of the various teams within the prison service. However, access to flexible working depends on where the staff member works and at what level in the service. Further, the underlying criteria for access to flexible working disadvantaged some staff.

It’s certain groups of people who can apply for formal flexi… They have to have children under whatever age or caring for a relative etc. Now as somebody who doesn’t have children, that sometimes annoys me because maybe I would like to work flexibly but I don’t qualify. (Laura, HQ, 11 years)

Similarly, certain pay grades came with greater responsibility and less flexible working, which deterred staff from applying for a promotion, since this would affect their work-life balance and consequent wellbeing.

If I went to a Band F and I remained [here], I wouldn’t be on flexi time and it has a big impact on the amount of leave you can take. (Gary, HQ, 18 years)

Mandatory training designed to fulfil statutory health and safety requirements is one of the keys to helping staff feel safe in their roles and to addressing the specific and sometimes distressing issues faced, such as complaints and fatal accident procedures. This reveals another overlap between wellbeing and professionalization policies. It is also about reducing corporate risk by making sure staff are, and remain, competent in their roles. Training is considered so important that the prison service has returned to having dedicated training officers. Respondents were positive about the training opportunities available, both specialised and more generic.

There’s ongoing training on just about everything, from health and safety to first aid, and everything in between… Even if you don’t do it within the prison service, they would send you elsewhere for training, ‘cos I’ve been to the Red Cross. (Glen, HQ, 23 years)

Supporting wellbeing within the prison service is easier in some locations than in others, and easier for some staff groups and levels than for others. Initiatives are often hampered by the requirements of the work and the working environment. Difficulties arise in releasing staff from their roles to fulfil training requirements. This is particularly the case with frontline officers.

You may need to get somebody on training but the job’s got to be done and if someone else went sick, you can’t have three of them absent. It can be difficult, but the reality is we get through it. (Felix, Prison, 18 years)

Staff recognise the links between wellbeing and professionalization support, but access can vary across the service. There is a need for flexibility in providing such initiatives and this requires special attention due to the secure nature of the prison context.

### Addressing the Underlying Tensions Inherent in Prisons

Prisons operate on behalf of society and are governed by statutory regulation, but they also have their own particular codes of conduct, designed to facilitate day-to-day functioning, and to keep staff and inmates physically, emotionally and psychologically safe. This combination of factors makes the prison setting a unique workplace. As one respondent put it:

It’s a particular world, you know, because it’s an institution and it’s about restricting freedoms … and that causes all sorts of problems and complications, which many of these policies are not obviously set up to engage with. (Dennis, Prison, 20 years)

The specialist training provided—on control and restraint, suicide prevention and care, and psychological manipulation—highlights the additional requirements for safety (in its broadest sense) in prisons. Consequently, the professionalization agenda has a strong focus on risk, personal security, health and wellbeing (compared with, for example, providing a quality service or maximising profit). Underpinned by the legal imperative to protect both staff and prisoners from harm, this further connects wellbeing and professionalization within the prison context.

It shows the importance of the [the specialist training] being there… we’ve actually got a manager specifically who programmes in training and makes sure that people’s competence levels are achieved. (Felix, Prison, 18 years)

Given the system’s broader evolution—from serving a “turnkey” (formal, distant and rule-enforcing) to more rehabilitative functions (flexible formality, more engagement, and greater situational interpretation of the rules and codes of conduct)—the realities on the ground mean that staff must switch between approaches depending on the situation they face. This was illustrated by one prison worker’s attitude towards acceptable language.

I got called a fucking troll… It was really funny but at the same time, there’s a limit to what you take and that was my limit. I’ve been called many things but I’m not getting called that! (Ginny, Prison, 2 years)

For the prisoners, this is a delicate dance; what is acceptable to one officer might not be acceptable to another. Equally, staff must remain alert to manipulative and coercive language that can easily slip into a seemingly innocent conversation. Clearly, the ongoing training is crucial, especially for staff who have specific prisoner-facing roles but who do not spend their entire time with prisoners.

It’s very easy to be caught up in a bit of banter one day and then the next you’re, you know, they’ve got you. (Dennis, Prison, 20 years)

Last week, we were away on conditioning training; how prisoners can condition you and kinda get you to do things for them. You get training how to watch [for that]. (Ingrid, Prison, 10 years)

No one on either side can let their guard down. However, the underlying tension in the working relationships between staff and prisoners does not necessarily always play out in predictable ways.

I’ve had one prisoner who wouldn’t back down… it was actually another prisoner that intervened and took my side and you start going: what’s just happened? (Ginny, Prison, 2 years)

While the media might focus on jails as violent places, respondents in this project spoke only of short-lived “skirmishes,” rather than of wholesale or ongoing violence.

Does it kick off? Very rarely. They’ve either been trying to hit another prisoner moving into a room and (we) pulled them away or they’ve attempted to assault an officer and they’ve been pulled away. Nothing to write home about. Just general skirmishes. (Ginny, Prison, 2 years)

However, if these skirmishes are not handled properly, the potential for escalation remains a source of significant tension, impacting both staff and prisoner wellbeing. Moreover, this has a cumulative effect, leading to more insidious forms of stress.

If, for example, there has been a serious incident, there are people who are there to coach the person through that afterwards and they get extra help. But I think sometimes maybe if there’s not an incident, there could be a series of events or dealing with a prisoner day to day that causes just as much stress as a one-hit operational problem… But whether people know how to tap into [the support given through the Employee Assistance Programme] or not, is different. (Laura, HQ, 11 years)

Given the considerable discretion required to maintain positive relationships and avoid “skirmishes,” the policy focus on supporting wellbeing also aims to mitigate this ongoing build-up of issues, on both sides, before critical incident point is reached. Although while most staff understand the reasons underpinning this more progressive approach, they remain “vulnerable,” not just to violence from inmates but also to the increased weight of policy requirements.

Years ago, before all the new policies … You’d probably have moved the prisoner—but not now. Everything’s got to be seen to be transparent and it is. But that doesn’t take away the vulnerability of people doing their job…

There is a lot of conflict regarding policies, you know. One minute you’ll be giving psychological manipulation training; how not to get caught up in conversations with prisoners giving away personal details, and the next minute, you’re trying to do suicide prevention in terms of engaging with prisoners… So in that sense, it’s a very fine line. (Dennis, Prison, 20 years)

These types of tension not only generate stress for prison workers but can also result in a lack of clarity for prisoners, damaging relationships. Other policies designed to improve health and wellbeing can themselves create idiosyncrasies and tensions that staff must manage. For example, it is difficult to promote smoking cessation in prisons when cigarettes still function as a currency and smoking remains a diversionary pleasure. The same applies to substance misuse policies (SPS 2019b). Such programmes can therefore fail to account for the complex prison environment and its specific differences from the outside world.

### The Impact of Professionalization

Professionalization in the SPS is rooted in the organisational values that all staff are expected to follow: a belief that people can change; respect for individuals, their needs and human rights; integrity in the application of high ethical, moral and professional standards; openness in working with others to achieve the best outcomes; courage to care regardless of circumstances; and humility in recognising that we can learn from others (SPS 2017). To be able to follow these ideals, staff require knowledge and understanding of their role, the skills and confidence to execute the role, and organisational support. This is provided through training, appraisal and a range of wellbeing initiatives—although changing professional expectations, an evolving prison environment, and increasingly multifaceted job roles mean that “professionalization” can be something of a moveable feast. Some interviewees had positive experiences of training.

If you are having difficulty with a project, you’ll get coached and mentored through something. They quite often give you something they know will be a challenge with the proviso you will get extra help to do it. (Laura, HQ, 11 years)

Prison workers often encounter complex scenarios, which may carry legal implications and require considerable discretion. As a result, much of the training and support available was felt to be somewhat abstract or generic. While this may be necessary in some respects, it left some interviewees feeling unsupported or inadequately trained.

The prison service takes the view that they’ll not comment on anything to do with prisoners or staff (incidents or accusations). That’s the legal side and in that sense, I think they leave some staff open to vulnerability in terms of support… until everybody [else] has done their piece of work, be it the police, the procurator fiscal, whatever. (Dennis, Prison, 20 years)

Given these limitations, many frontline workers distinguished between what is formally trained for, and more informal/pragmatic responses, which may present a different set of solutions that are more effective “in the moment.” Navigating between organizational policy and everyday realities is a challenge for many street-level bureaucrats, which can lead to considerable stress if organizational messaging is unclear (Zacka 2017).

At the formal end of the spectrum, a suite of training programmes and policies exist. The *SPS Corporate Plan 2019/2022* outlines an agenda centred on further education, training and continuing professional development, a renewed focus on peer support, and an ultimate aim to develop leadership capability among all staff (SPS 2019a: 22). However, interviewees’ experiences and perceptions of professionalization tended to focus on the more coercive aspects and performance management systems, such as “Charter for Help,” which is:

…a structured programme whereby if that person is not performing in the job role as they should be… It’s almost a step-by-step plan of how they’re helped and coached to get to the standard that’s required of them. (Laura, HQ, 11 years)

The more informal concepts of professionalism were defined by, and discerned in, day to day working practices, which became embedded in staff attitudes and behaviours. For example: in the rights of passage for new recruits within the prison.

You get staff in every organisation who are nae interested [until you] prove yourself… I’ve had prisoners come up to me and start bawling and shouting at me and you’re like, “do me a favour, go away, put your pleasant head on and come back to me and then I’ll speak to you”… You can see the [other staff] behind you, they are itching to get in there and you’re like, “no, no he’ll learn he’s no gonna speak to me like that”… See, the minute you’ve had your first incident and stuff like that, you’ve proved yourself to them. (Ginny, Prison, 2 years)

Or dealing with staff shortages and covering colleagues who may be off sick—a sense of solidarity develops, especially among close-knit teams working under pressure (Vogl 2009).

Staff rally round… We’ve got a staff member who was off sick, who was getting to the stage where [they] were no gonna get any wages. Staff said “right we’ll cover [their] post” and they all done extra hours to cover [that person]’s post. (Felix, Prison, 18 years)

These more “informal” practices filled the gaps in formal policies and training. As with the wellbeing initiatives, motivation was a key factor. Staff must be able and want to do the job if more formal policies and training are to work as supports and not as tick-box exercises. Increasing work pressures and fluctuating relationships are a part of the prison context, and these have a significant effect on “attitudes” and motivation to undertake training and to develop professionalism.

We can do a number of things but at the end of the day… If you are looking to get something out of someone, that person has to actually come with the right attitude and without that right attitude, nothing’s ever going to get done … It’s the maintaining of motivation that’s the difficult thing. Part of the reason for that is the high prisoner numbers; more jobs to do and less time to do them. (Dennis, Prison, 20 years)

The intersection between professionalization and wellbeing is also reflected in a significant increase in prison health and safety policies, such as Management Of Risk in Extreme Circumstances (MORE), Management of an Offender at Risk due to any Substance (MORS) and Organised Prisoner Networks (OPN). The role of prison staff has become much more complex, even in the relatively unchanging physical arena of security and safety. The professionalization training seems to be well installed; prison workers know what to do under pressure—but the link with vulnerability and wellbeing, both on the ground (the threat of violence) and in policy terms (administrative or legal retribution) cannot be ignored. One participant observed how their high level of training could also serve to magnify tension, leading to increased stress. Here, an interesting link was made between professionalization and wellbeing:

Well, the stress part is probably waiting for something to happen. Once it happens, we’re very good at dealing with what happens. It’s the atmosphere and the build-up and the tension and the unknowing of what’s going to occur that causes the stress. But once anything kicks off, then we are sort of trained, or programmed, in order to deal with that situation and we usually deal with it very well. (Dennis)

The “moveable feast” of professionalization, combined with the broader challenges of understaffing and high stress, have made it difficult to retain both newly trained and experienced staff (Auditor General for Scotland 2019: 9). Now that new prison officers are expected to work until age 68, the need to understand the realities of career development and to enact an effective professionalization agenda are even more pressing. This would be reflected in a more inclusive approach to policy development and design, as noted by one front line prison worker:

Perhaps people should actually come and speak to the staff prior to going and making their policies and agreements… To see how it works in the real [prison] world out there… There’s a reason why staff do things in certain ways, because the world in here is different to the world out there and they might not see that. (Dennis)

## Discussion

These findings highlight *some* of the complexities surrounding the ‘whole prison approach’, particularly in developing and implementing wellbeing policies that are inextricably linked with those on safety and professionalization. This interwovenness generates similar challenges and dilemmas to those faced by street-level bureaucrats, except that the complex and mostly self-contained prison environment can magnify such problems. The growing prison population and consequent pressures on prison workers make this an issue worthy of attention.

### Wellbeing Support Mechanisms Need to Be Fully Accessible

The increasing focus on health and wellbeing in many organisations has generated a raft of policies and mechanisms designed to deliver a more “caring” organisational approach. This includes recognition of and support for more complex stress-related issues and/or substance misuse. In Scottish prisons, many wellbeing initiatives are driven by the Healthy Working Lives Award programme, which places the onus on staff to take responsibility for their health and wellbeing. As a result, while respondents agreed that there were theoretically plenty of opportunities for supporting a better work-life balance, many struggled to properly engage with these. For example, frontline staff (and managerial staff with no job-share counterpart) had no access to flexible working, despite such policies also being aimed at improving overall wellbeing. Similarly, shift work was largely incompatible with healthy eating programmes (staff on night shifts made do with vending machine food), or activities that took place at times that were hard to juggle with shifts. If increasing staff wellbeing is to be self-motivated, then wellbeing initiatives need to be more accessible and inclusive.

### Staff and Prisoners’ Wellbeing Are Linked: Balancing Safety and Care

Prisoner and prison worker mental health are linked (Beynon and Drew 2005). The logic is straightforward; prisoners who feel valued and respected, and who have positive relationships with staff, are less likely to be violent towards staff, who in turn feel safer, with lower stress levels (Misis et al., 2013). Basic physical and mental health support for both inmates and staff is embedded into the ‘whole prison approach’ but more progressive, preventive or complex approaches are hampered by high prisoner intake and low staff numbers (Schmidt et al., 2017; Council of Europe 2019). For example, for prison staff, maintaining a safe psychological distance while also trying to engage with prisoners as part of a rehabilitative package echoes the challenges faced by psychological therapists but without the depth of training. These types of delicate relationships are “central to the running of prisons” (Liebling, Price, and Shefer 2011: 200).

Balancing safety and care while maintaining positive relationships requires experience, confidence and the ability to use discretion in a positive enabling manner. Such qualities are beginning to filter into prison officer development agendas, for example, through the embedding of “affective learning” into training regimes (Morrison 2019). Experienced prison workers had noticed the increasing emphasis on—and investment in—more merged corporate safeguarding, training and wellbeing policies. The fluid and responsive configuration of supporting health, care, rehabilitation and safety means that discretion may operate as a moderator between corporate approaches and daily realities. The SPS links these issues in its wellbeing and professionalization policies but our data suggest that more can be done to account for frontline workers’ experiences in different roles across the service.

### Professionalization Is a Two-Way Street

The rise in HR-related procedures such as appraisal processes, performance management, KPIs and longer-term career support, contributes to an architecture of professionalization that was predicted nearly 30 years ago.

The basic task of the prison officer has not changed in 150 years. It remains that of the secure custody of the prisoner and this is inevitably so … [However], we may now be at the beginning of a fundamental reassessment of how the prison officer carries out this task … in the not too distant future the prison officer might properly be described as a professional worker (Coyle 1991: 242–43).

Liebling, Price, and Shefer (2011) argue that professionalization emerges from a culture of managerialism, noting that performance management and the use of “targets” reflects a shift towards privatisation (two of Scotland’s prisons are privately managed under contract to the SPS) and neoliberal efficiency trends. They observe that for frontline staff, the core functions of the job remain unchanged but since much of the officers’ primary activity is centred on their relationships with prisoners, “targets” can be difficult to quantify, and some staff believe that KPIs “fail to capture the “essence” of their work” (ibid.: 202). Increasing use of metrics and quantization, also an issue in healthcare (Brown and Baker 2012) and education (Lakes and Carter 2011), remains problematic for workers whose job roles require any form of discretion, and the closed system of the prison seems to increase this pressure.

“Professionalization” within the prison service has for some staff created a more complex and nuanced role, where attitudes and values are increasingly important to delivering the service the SPS aspires to. Morrison (2019: 26–27) argues that “the enactment of even the most neutral policies, tasks and processes will be shaped by the attitudes, beliefs and emotions of the groups and individuals in question.” This is critical, given that the meaning of “being professional” is mediated by both formal policy and the everyday realities of working in prisons. Behaviours that outsiders might consider “unprofessional” might, in the prison context, be considered professional by prison staff. For example, our study revealed instances of staff developing their own forms of discretion; allowing other prisoners to “intervene” in skirmishes or stepping back to allow colleagues to “prove” themselves, and often these solutions helped to ease everyday stresses for both officers and prisoners.

The increasing number of policy initiatives seems to be developing without regard to the impact on staff resources and by extension (ironically) staff wellbeing. There are only so many hours available to accommodate work and mandatory training, and to update on new and complex policy developments and their impacts throughout the system. *Forward Together*, the partnership agreement between the SPS and its Trade Union Side (SPS 2016a: Annex E) made significant reference to providing the flexibility and resources to make training accessible but this was not consistent with many of the comments in our interview data. Introducing more policies without fully taking into account the tensions and resourcing pressures within the system risks a backlash from prison staff, as evidenced by the SPS Officers’ rejection of the POPP programme (SPS 2019b: 2).

### Tensions Between Care and Safety Reveal Broader Issues of Control

The “taming” of state-enacted violence against prisoners has shaped modern regimes designed to safely control violent individuals (Foucault 1977), giving rise to frameworks based on selective risk management (Genders and Player 2014), as well as more economically rational policy agendas such as the “whole prison approach.” This type of “systems” approach goes hand in hand with broader neoliberal ideas of control (Wacquant 2009). In this framing, prison workers’ discretionary roles become subverted; training on bureaucratic responses to violent incidents articulates a threat of administrative retribution, forcing staff to police themselves. Prison staff are therefore controlled from above and below; by prisoners, through the persistent threat of physical and psychological violence, and by the increasingly corporatized institutional apparatus, through training, appraisal and performance management. This leads to a dilemma: wellbeing and professionalization agendas aim to enhance positive relationships, good decision-making and a rehabilitative ethos, but simultaneously exert a bureaucratic control, suppressing discretionary decisions and making staff hyper-aware of the limitations of their roles.

Foucault uses the term “disciplinary career” to refer to both the controllers and the controlled in institutions across society (Adam 2015: 54–55). We acknowledge this antecedent theory of social control lightly here; while it dovetails with the present situation in which underlying tensions and the threat of violence remain key policy drivers, we are not endorsing a corrective position of increased discipline of prisoners.^5^ The SPS policy approach, with its dual focus on training and wellbeing, appears a well-intentioned attempt to create a more concrete version of the disciplinary career, potentially raising the status of prison worker roles. Policy design should start from a position of recognising this dilemma to better understand the role of discretion, other tensions faced by frontline workers, and consequently their wellbeing and training needs.

The issues of discretion and officers’ wish to participate in decision-making raise important questions about the role of the modern prison officer, the training officers receive and the management of officers in their work … The “professionalization” of prison officer work and the more demanding nature of modern prison regimes require, on the one hand, a move away from rigid organisational control, yet on the other, carefully managed systems of guidance and accountability. Hands-on leadership is essential in such a complex working environment (Liebling, Price, and Shefer 2011: 202).

## Ways Forward

The “whole prison approach” and its attendant raft of professionalization and wellbeing initiatives has highlighted the difficulty in developing useful *and* usable policies in a complex and fragmented setting. Our findings suggest a number of potential ways forward for policy design:

• Involve prison staff in policy design. Calls for more “on the ground” involvement in policy development are common and our study indicated clearly that the voice of frontline staff is missing from the policy design process. Relatively simple solutions, such as suggestion boxes and more two-way communication with management, might enable more pragmatic results than the somewhat unidirectional staff wellbeing survey. It is worth revisiting the quote from Dennis: “Perhaps people should actually come and speak to the staff prior to going and making their policies and agreements…”
• Recognise interdependence between prisoners, between staff, and between prisoners and staff. This reveals a strong sense of solidarity. (Morgan and Pulignano, 2020: 18). highlight how solidarity “partakes of moral, political and performative elements that are underpinned and reinforced by a shared work context,” and that new forms of solidarity emerge according to evolving organisational infrastructure and an institutional frame. Involving frontline staff and prisoners in blended initiatives would capitalise on prison-specific solidarities, potentially enabling a more horizontal or even co-designed policy approach. This would encompass a wider range of experience and knowledge, which could be tailored to different prison contexts such as high-security, long term, hospital facilities, YOIs and so on.
• The diverse range of specific training courses available (and uneven accessibility) creates a somewhat ‘pick and mix’ approach to career development, making it difficult to create overarching and core knowledge. A more coherent professionalization “menu” might include: shadowing of various roles where possible; greater acknowledgment of everyday tensions; increased humanities content to develop deeper understandings of the social role of prisons; and more empowering training around decision-making and discretion.
• Flexible working and inclusion. While heavily influenced by external factors such as resourcing, human rights legislation, responding to evaluations, or aiming to improve metrics around mental health, the ethos of inclusion must be embedded into all future planning and policy design. Policies that are only partially accessible will fail to bring about positive change across the service and can make existing tensions more entrenched.

The Scottish Prison Service promotes a dual wellbeing and professionalization agenda, which has been generally well received by employees. However, it fails in some respects to account for the realities of prison life, itself a specific and rarefied context that is comparatively under-researched in the work and professions literature. We interviewed a particular stratum of prison service workers whose understanding of both centralized/policymaking and frontline perspectives enabled a deeper insight into the issues arising. Due to the small sample size, our primary data should be considered only in terms of the specific circumstances of the researched group and we caution against generalising beyond this. The policy data however enables a more concrete set of background assumptions, which apply across the Scottish prisons context. Combining these two sources, alongside wider literature, reveals a need for more detailed study across the SPS, asking more specific research questions and accounting for local policies or facilities.

A more integrated wellbeing, training and professionalization agenda that recognises the unique nature and challenges of the prison environment would help to alleviate some of the tensions identified in this study, improving life for both prisoners and prison workers. With this in mind, we endorse the current integration of wellbeing and professionalization policies but believe more can be done to implement these in a manner consistent with the realities of prison life.

## Data Availability

The raw data supporting the conclusions of this article will be made available by the authors, without undue reservation.

## Footnotes

1 Most of these studies are from western countries. Akoensi (2018) found that Ghanaian prison officers’ work impacted their ability to commit to family responsibilities—and this was a more significant problem in Ghana’s more collectivist and family-oriented culture.

2 The data collected predates the Covid-19 pandemic, which has had a significant impact on the pressures faced by all prisons and can further complicate staff/prisoner dynamics. However, these factors do not alter the core argument presented here.

3 The professionalization agenda taken forward initially through the Prison Officer Professionalization Programme (POPP). However, in 2018, the Scottish Prison Officers Association rejected that structure (Hogg 2018) and a new corporate plan 2019–22 was put in place incorporating some of the building blocks of POPP (SPS, 2019a).

4 The NHS Five Year Forward View refers to the English context but its broad focus on prevention, community and public health is shared by health organisations across the United Kingdom.

5 It is worth disaggregating the more directly Foucauldian perspective, policy as a form of coercive control, from the perspective offered earlier in this article, effective training to protect against other disciplinary threats. Arguably, these threats come from the same bureaucratic system, so a Foucauldian analysis could be applied. However, this article aims to report more surface-level findings and emergent issues, with a view to making practical recommendations.

## Authors’ Note

The raw data supporting the conclusions of this article is lodged with Qualidata as per the final requirements of the original ESRC grant.
